# CFIm25-regulated lncRNA *acv*3UTR promotes gastric tumorigenesis via miR-590-5p/YAP1 axis

**DOI:** 10.1038/s41388-020-1213-8

**Published:** 2020-02-17

**Authors:** Kai Liu, Ben-Jun Wang, WeiWei Han, Chun-Hua Chi, Chao Gu, Yu Wang, Xiaohai Fu, Wei Huang, Zhiguo Liu, Xilin Song

**Affiliations:** 1 Department of Gastrointestinal Surgery, Shandong Cancer Hospital and Institute, Jinan, China; 2 Department of Anorectal Surgery, Affiliated Hospital of Shandong University of Traditional Chinese Medicine, Jinan, Shandong 250014 China; 3 Department of Radiology, Affiliated Hospital of Shandong University of Traditional Chinese Medicine, Jinan, Shandong 250014 China; 4 Department of Intensive Care Unit of Shouguang People’s Hospital, Shouguang, Shandong China; 5 Department of Radiation Oncology, Shandong Cancer Hospital and Institute, Jinan, China; 6 PET-CT Center, Shandong Cancer Hospital and Institute, Jinan, China

**Keywords:** Biochemistry, Gastric cancer

## Abstract

Accumulating evidences indicate that 3ʹUTR of the coding gene can act as crucial regulators in gastric cancer (GC). However, the detailed mechanisms and responsive targets are not well established. Here, we found that *acvr1b* gene 3ʹUTR (*acv*3UTR) was elevated in GC tissue, the expression of which was significantly correlated with advanced pTNM-stage and poor outcome in clinical patients. Forced expression of *acv*3UTR promoted GC cells growth in vitro and in vivo. Mechanistically, our results suggested that *acv*3UTR functioned as an oncogenic competing endogenous RNA via sponging miR-590-5p and enhancing YAP1 level. Tumor suppressor miR-590-5p was a molecular module in *acv*3UTR regulatory axis, the forced expression of which led to impairing of oncogenic potential of *acv*3UTR. The positive correlation of *acv*3UTR and YAP1 expression, and the negative correlation of *acv*3UTR and miR-590-5p expression, were verified in GC patients. Moreover, CFIm25 was identified as a key regulator contributing to *acv*3UTR aberrant expression in GC binding to UGUA-264 motif. Overall, our finding defines a mechanism for understanding the potential role of *acv*3UTR transcription in GC tumorigenesis, and indicates a correlation between 3ʹUTR *trans*-regulatory effect and GC development.

## Introduction

Gastric cancer (GC) is an aggressive malignancy with a globally increasing incidence, especially in eastern Asian countries [1, 2]. Despite the advances in diagnostic and therapeutic strategies, the clinical outcomes of GC patients have not improved because of late diagnosis, tumor relapse, and drug resistance [3]. Therefore, deciphering the genetic and epigenetic alterations, especially the molecular mechanisms underlying the tumorigenesis and progression of GC, will benefit the identification of novel diagnostic biomarkers and development of new therapeutic strategies.

Most of the human genomes are transcribed into noncoding RNAs [4]. The aberrant expression and deficiency or mutation of these noncoding RNAs played important roles in cancer [5]. Noncoding RNAs, especially long noncoding RNA (lncRNA), took part in a variety of biological processes, including chromatin interaction, transcription regulation, mRNA post-transcriptional regulation, and epigenetic regulation [6]. Hundreds of dysregulated lncRNA were identified, the aberrant expression of which was frequently associated with GC clinical pathologies [7]. Recently, the competitive endogenous RNA (ceRNA) hypothesis provides new insights into the function of lncRNA [8]. By competing for microRNAs (miRNAs), lncRNA indirectly upregulates the expression of target genes. In GC, the lncRNA-associated ceRNA network has been comprehensively analyzed, and some lncRNAs were identified as oncogenic tumor-suppressive ceRNAs [9, 10]. For instance, LncRNA BC032469 acted as a ceRNA for miR-1207-5p to upregulate the expression of hTERT, and promoted proliferation [11]. MT1JP functioned as a tumor-suppressor ceRNA by competitively binding to miR-92a-3p to active FBXW7 expression, or by competitively binding to miR-214-3p [12, 13].

Besides lncRNA, other types of RNA transcripts can also function as ceRNAs, such as 3ʹUTRs [14, 15]. Evidences suggested that 3ʹUTR usually plays an important role as lncRNAs [16, 17]. It is clear that mRNA 3ʹ-end formation is subject to dynamic regulation under diverse physiological conditions. Usually rapidly proliferating and transformed cells preferentially express mRNAs with shortened 3ʹUTRs [18, 19].

CFIm25 (cleavage and polyadenylation-specific factor 5, 25-kD subunit) is a key regulator of 3ʹUTR formation in mammalian cells. The CFIm25 dimer is clasped on opposite sides by two CFIm68 RRM domains [20]. CFIm25 can bind UGUA sequences in pre-mRNA, and promote the synthesis of longer mRNA isoforms [21]. Downregulation of CFIm25 in multiple human and mouse cell lines leads to 3ʹUTR shortening in hundreds of genes, and a consequent increase in protein levels of a subset of those genes [22]. CFIm25 expression inhibition in glioblastoma cells enhances their tumorigenic properties, and increases tumor size, whereas CFIm25 overexpression reduces these properties and inhibits tumor growth [22]. However, the function and regulatory mechanism of CFIm25 in GC is largely unknown.

Acvr1B (activin receptor type-1B, also known as Alk4) is a transmembrane protein that in humans is encoded by the *acvr1b* gene, which is essential for signaling transduction of activin signal pathway [23]. ACVR1B together with ACVR2A or ACVR2B forms a receptor complex for activin, a member of the transforming growth factor-β superfamily [24]. ACVR1B becomes activated upon ligand binding, and in turn phosphorylates and activates Smad proteins [24]. ACVR1B has a well-established role in vasculogenesis. Loss of function of ACVR1B is a primary cause of autosomal-dominant vascular dysplasia syndrome [25], while increase of ACVR1B induces blood vessel branching [26]. In cancer studies, there are several researches showing different roles of ACVR1B based on different cancer types, such as tumor-suppressor role in pancreatic cancer [27, 28], oncogenic roles in small-bowel adenocarcinoma [29], prostate cancer [30], and leukemia [31]. To our knowledge, there is no functional study of ACVR1B in GC.

Here, we found that the 3ʹUTR of acvr1b can function as a lncRNA. We named it as *acv*3UTR. Our study demonstrates that *acv*3UTR is a CFIm25 target gene, which is frequently upregulated in GC samples, and played an oncogenic role by functioning as an oncogenic ceRNA via sponging miR-590-5p and activating YAP1 expression.

## Results

### Acvr1b is frequently upregulated in GC tissues and associated with GC clinicopathologic factors

**Fig. 1:**
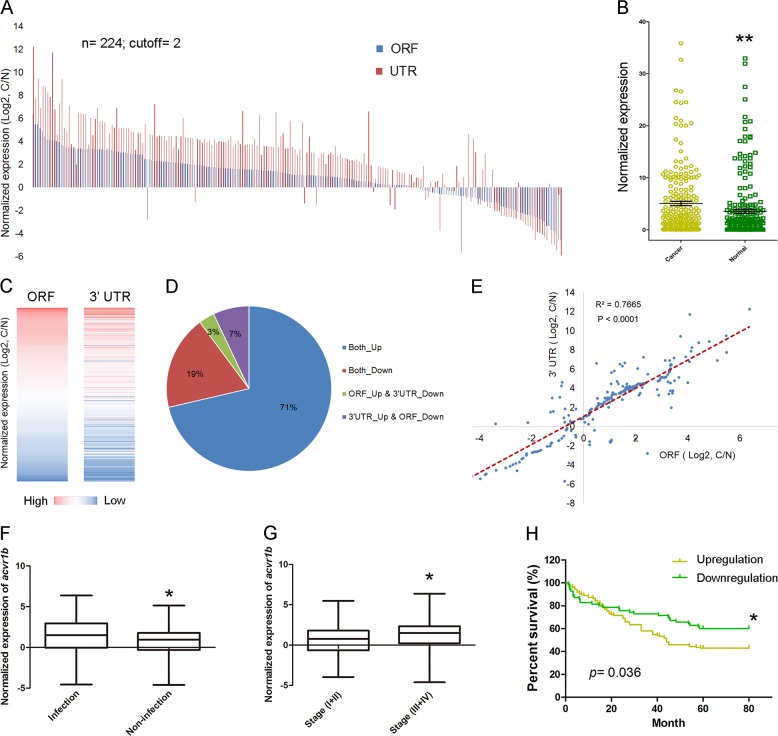
Acvr1b upregulation is associated with advanced progression and poor prognosis in GC. **a** The expression level of acvr1b ORF and 3ʹUTR was validated in 224 paired GC samples with qRT-PCR. C: GC tissues. N: GC-adjacent tissues. GAPDH was the internal control. **b** Normalized expression of acvr1b in 224 paired GC samples with qRT-PCR. **c** The heatmap showed the expression of ORF or 3ʹUTR from the same patient (total 224 patients). **d** Pie chart showed that 71% of patients showed both ORF and 3ʹUTR upregulation. **e** Correlation of acvr1b 3ʹUTR and ORF expression. **f** The relative level of acvr1b in the patients with or without *Helicobacter pylori* infection. **g** The relative level of acvr1b in the early pTNM-stage (I + II) and advanced pTNM-stage (III + IV) patients. **h** The relation between overall survival rate of GC patients and the acvr1b level was analyzed by Kaplan–Meier survival curve. *P < 0.05; **P < 0.01; ***P < 0.001 (paired *t*-test, two tailed).

To study the association of *acvr1B* with the occurrence of GC, we assessed the expression of *acvr1B* in 224 clinical GC patients. As a result, the expression of *acvr1B* was significantly upregulated in 162 cases (72.3%) compared with noncancerous adjacent tissues using a twofold expression difference cutoff (*p* = 0.0092) (Fig. 1a, b). Interestingly, patients with *Helicobacter pylori* infection exhibited significantly higher *acvr1B* level compared with noninfection patients (*p* = 0.0418) (Fig. 1f, Table S1). These results suggested that acvr1b could play roles in GC, especially in GC patients with *Helicobacter pylori* infection.

To further analyze the association of acvr1b expression to GC carcinogenesis, we compared acvr1b expression levels with the clinicopathological features in patients with primary GC (Table S1). As a result, the *acvr1B* level was significantly lower in the early pTNM-stage (I + II) compared with advanced pTNM-stage (III + IV) tumors (*p* = 0.0426) (Fig. 1g, Table S1). Kaplan–Meier survival analysis indicated that the overall survival rates among patients with GC were significantly higher in patients with low *acvr1B* level than in those with high *acvr1B* level (Fig. 1h). These results further demonstrated the possibility that *acvr1B* functions as an oncogene in GC.

### *Acv*3UTR played roles as an oncogenic RNA

**Fig. 2:**
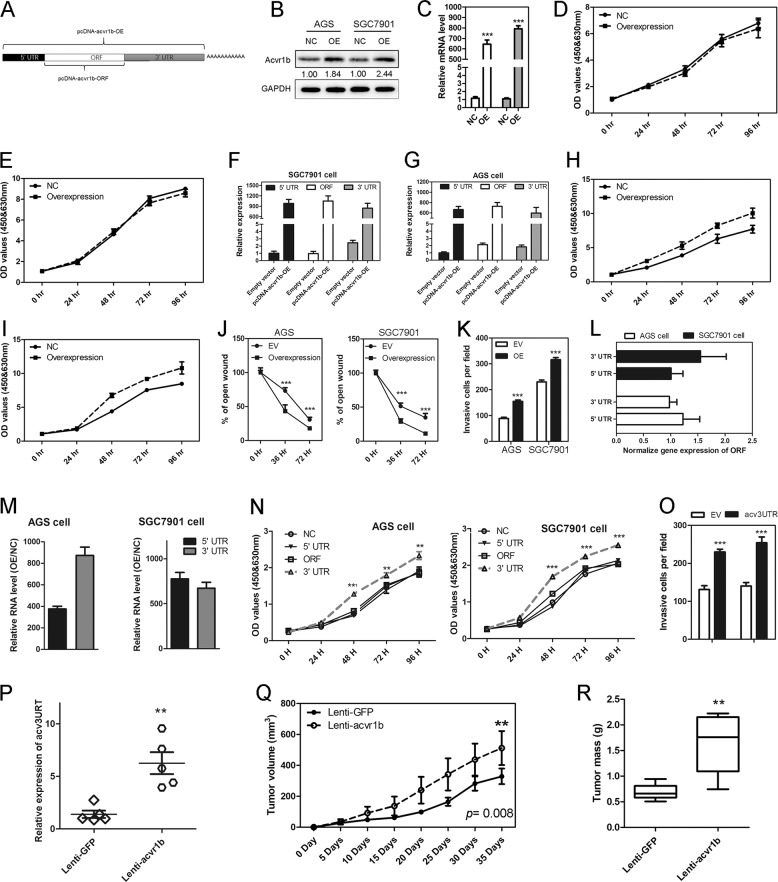
Acv3UTR promotes GC tumorigenesis in vitro and in vivo. **a** Acvr1b overexpression strategy. **b** Acvr1b ORF overexpression increased protein level. **c** Acvr1b ORF overexpression increased RNA level. **d** Acvr1b ORF overexpression did not affect proliferation of AGS cells. **e** Acvr1b ORF overexpression did not affect proliferation of SGC7901 cells. **f** Full-length acvr1b overexpression increased 5ʹUTR, ORF, and 3ʹUTR expression in AGS cells. **g** Full-length acvr1b overexpression increased 5ʹUTR, ORF and 3ʹUTR expression in SGC7901 cells. **h** Full-length acvr1b overexpression promoted proliferation of AGS cells. **i** Full-length acvr1b overexpression promoted proliferation of SGC7901 cells. **j** Full-length acvr1b overexpression promoted GC cell migration. **k** Full-length acvr1b overexpression promoted GC cell invasion. **l** Acvr1b 5ʹUTR or 3ʹUTR overexpression did not change the ORF level. **m** Acvr1b 5ʹUTR or 3ʹUTR overexpression efficiency. **n** Acv3UTR overexpression promotes GC cell proliferation. **o** Acv3UTR overexpression promotes GC cell invasion. **p** Acv3UTR level in xenograft tumor detected by RT-PCR. **q** Tumor volume in acv3UTR-overexpression group (Lenti-acvr1b) or control group (Lenti-GFP). **r** Tumor mass in *acv*3UTR-overexpression group (Lenti-acvr1b) or control group (Lenti-GFP). NC negative control. OE overexpression. *P < 0.05; **P < 0.01; ***P < 0.001 (paired *t*-test, two tailed).

To determine the potential regulatory function of acvr1b in GC, we cloned acvr1b ORF region into pcDNA3.1 plasmid (Fig. 2a), the transfection of which enhanced both Acvr1B protein and RNA level (Fig. 2b, c), but exhibited no regulatory role in GC cell proliferation (Fig. 2d, e). However, full-length sequence overexpression of Acvr1b, which enhanced expression of acvr1b ORF, 5ʹUTR and 3ʹUTR (Fig. 2f, g), significantly promoted GC cell proliferation, migration and invasion (Figs. 2h–k, S1A, and S1B). Based on these results, we hypothesized that it was the 3ʹUTR or 5ʹUTR playing the oncogenic RNA roles on GC cell proliferation. We then overexpressed acvr1b 5ʹUTR or 3ʹUTR separately, the overexpression of which did not change Acvr1B ORF RNA level or Acvr1B protein level, but only upregulated 5ʹUTR or 3ʹUTR (Figs. 2l, m, S1C, and S1D). As a result, 3ʹUTR (*Acv*3UTR) overexpression significantly enhanced GC cell proliferation and invasion (Figs. 2n, o, and S1E).

Xenograft node mice model was performed to evaluate the function of *acv*3UTR on tumor growth in vivo by subcutaneous injection of SGC7901 cells with stable *acv*3UTR overexpression (lenti-acvr1b) or GFP overexpression (lenti-GFP) (Fig. S1F). Acv3UTR overexpression efficiency was validated by qRT-PCR (Fig. 2p). Compared with negative control, lenti-acvr1b mice exhibited significantly larger tumor volume (Figs. 2q, S1G, and S1H) and significantly higher tumor weight (Fig. 2r).

We also designed primers specifically detecting *acv*3UTR expression in clinic GC samples. As a result, *acv*3UTR expression was enhanced in 169 cases (75.4%) compared with noncancerous adjacent tissues (Fig. 1a), which showed the same expression pattern as acvr1b ORF (Fig. 1c–e).

Taken together, these results indicated that *acv*3UTR played roles as an oncogenic RNA.

### *Acv*3UTR acted as a sponge to sequester miR-590-5p

Noncoding RNAs can serve as ceRNAs that regulate other gene expression through miRNA-dependent crosstalk [32, 33]. Hence, we sought to determine a possibility that acvr1B-regulated GC cell proliferation functions as a ceRNA associated with miRNAs.

**Fig. 3:**
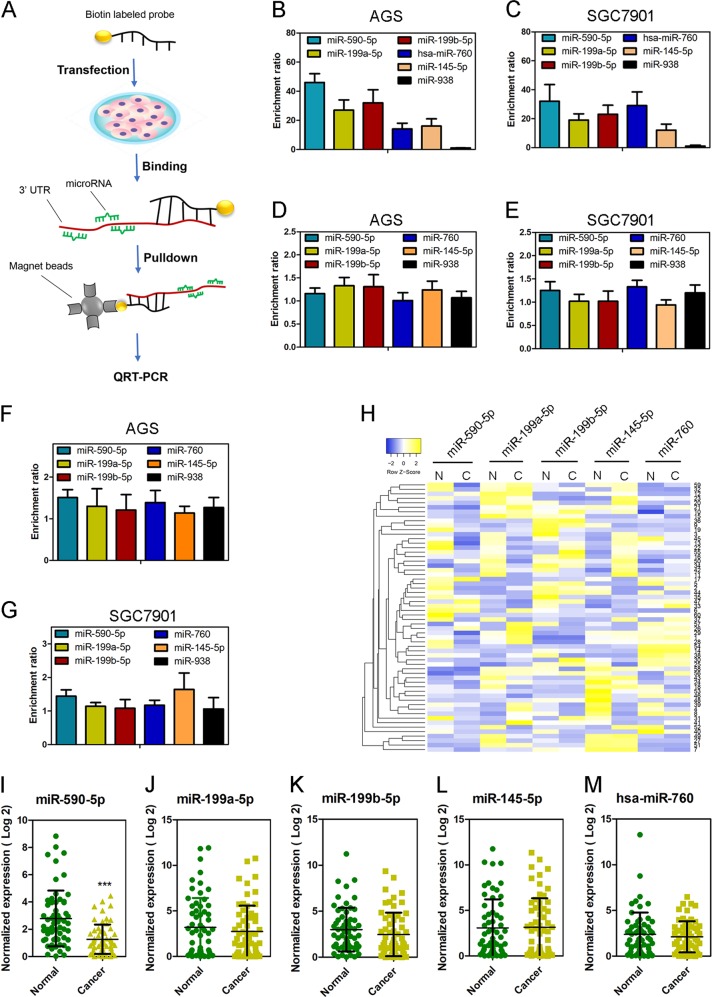
Acv3UTR acts as a sponge to sequester miR-590-5p. **a** Schematic diagram of RNA-pulldown study. **b**, **c** Five enriched microRNAs by acv3UTR pulldown. MiR-938 was used as a negative control. The five acv3UTR-enriched microRNAs were not enriched by acvr1B 5’UTR (**d**, **e**) or ORF (**f**, **g**) pulldown. **h** Heatmap showed the expression of five acv3ʹUTR-enriched microRNAs in 60 pairs of GC clinic samples with high acv3UTR expression. The expression of miR-590-5p (**i**), miR-199a-5p (**j**), miR-199b-5p (**k**), miR-145-5p (**l**) and miR-760 (**m**) in 60 pairs of GC clinic samples. ***P < 0.001 (paired *t*-test, two tailed).

First, the web tool Targetscan [34] was used to predict the potential acv3UT-binding miRNAs, and 43 miRNAs found had conserved sites in *acv*3UTR (Table S2). Second, RNA pulldown and qRT-PCR was carried out using biotin-labeled DNA probe to investigate the physical interaction between *acv*3UTR and miRNAs (Fig. 3a). As a result, five miRNAs were significantly enriched (enrichment ratio>tenfold), and miR-590-5p was mostly enriched, whereas the enrichment of negative control miR-938 showed no difference (Fig. 3b, c). No enrichment of these miRNAs was found when performing RNA pulldown using probe-targeting 5ʹUTR or ORF (Fig. 3d–g), suggesting that the physical interactions of these five miRNAs were specific for acv3UTR. MiR-590-5p was significantly downregulated in GC tissue, while no significant difference was found for other miRNAs (Fig. 3h–m). Based on these results, we chose miR-590-5p for further study.

### MiR-590-5p functions as a tumor suppressor by targeting YAP1

**Fig. 4:**
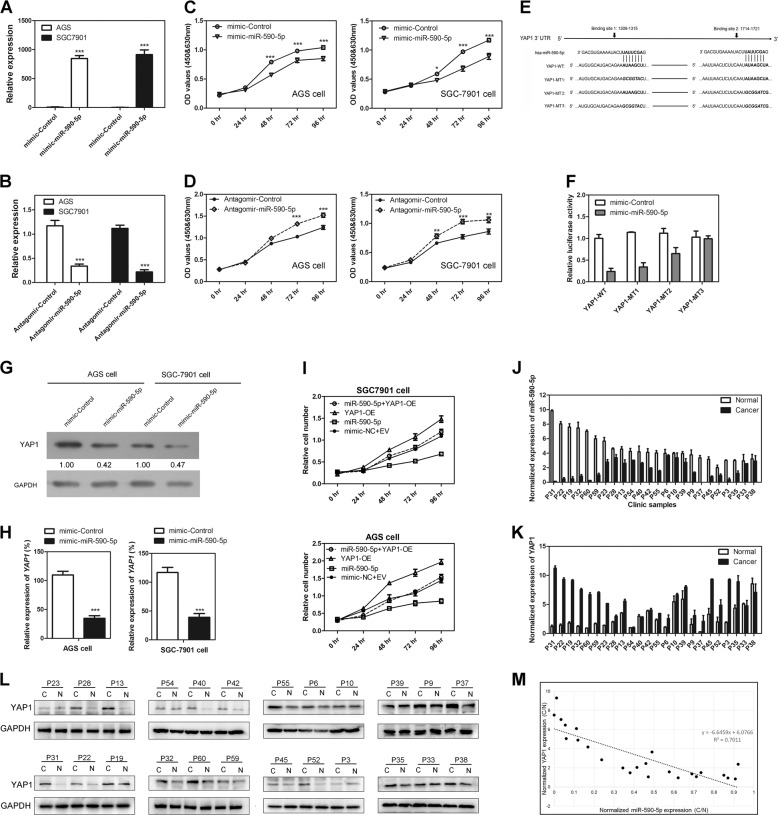
MiR-590-5p functions as a tumor suppressor in GC by targeting YAP1. **a**, **b** MiR-590-5p was successfully overexpressed or knocked down in GC cells. **c** MiR-590-5p overexpression suppressed GC cell proliferation. **d** MiR-590-5p knockdown promoted GC cell proliferation. **e** The potential target sites of miR-590-5p in YAP1. **f** Luciferase assay demonstrated miR-590-5p targeting both binding site 1 and binding site 2 of YAP1. **g**, **h** miR-590-5p inhibited YAP1 on both protein level and RNA level in GC cell. **i** Rescue experiments showed that the inhibition effect of miR-590-5p on GC cell proliferation can be rescued by YAP1 overexpression. **j** MiR-590-5p was downregulated in GC tissue in clinic samples. **k** YAP1 mRNA was upregulated in GC tissue in clinic samples. **l** YAP1 protein was upregulated in GC tissue in clinic samples. **m** MiR-590-5p expression showed negative correlation to YAP1 expression. *P < 0.05; **P < 0.01; ***P < 0.001 (paired *t*-test, two tailed).

We then investigated the function of miR-590-5p during GC tumorigenesis. First, we modified miR-590-5p expression in GC cells (Fig. 4a, b), finding that miR-590-5p overexpression significantly inhibited GC cell proliferation while miR-590 knocking down enhanced GC cell proliferation (Fig. 4c, d), suggesting a tumor suppressor role of miR-590-5p in GC cells.

Second, we explored the regulatory mechanism of miR-590-5p in GC. YAP1 has been reported as a target of miR-590-5p in colorectal cancer [35]. There are two potential binding sites in 3ʹUTR of YAP1. We produced a luciferase construct of YAP1 wild type (YAP1-WT) and mutated form (YAP1-MT1, YAP1-MT2, and YAP1-MT3), and dual-luciferase reporter assay was used to verify the interaction between miR-590-5p and YAP1 (Fig. 4e). Cotransfection of miR-590-5p with Acvr1b-WT leaded to significantly lower luciferase activity, while repression effect of miR-590-5p on the luciferase activity was partially impaired by single binding site mutation (YAP1-MT1 or YAP1-MT2) and totally impaired by dual sites mutation (Fig. 4f), indicating both sites were true miR-590-5p binding sites. Enforced expression of miR-590-5p significantly decreased YAP1 protein and RNA level, indicating that YAP1 was a direct target gene of miR-590-5p, negatively regulated by miR-590-5p in GC cells (Fig. 4g, h).

At last, rescue experiments were performed to confirm the regulatory role of miR-590 on YAP1. As shown in Fig. 4i, the inhibition of miR-590-5p on GC cell proliferation was rescued by YAP1 overexpression. We also determined the YAP1 RNA level and protein level in top 24 clinic patients with miR-590-5p downregulated in GC tissues (Fig. 4j). In contrast to lowered miR-590-5p expression, YAP1 showed significant enhanced expression in GC tissue on both RNA and protein levels (Fig. 4k, l). The expression of YAP1 exhibited negative correlation to miR-590-5p (Fig. 4m). Taken together, these results confirmed that miR-590-5p suppressed GC cell proliferation by negatively regulating YAP1 in GC.

### *Acv*3UTR promoted GC cell proliferation by competing miR-590-5p

**Fig. 5:**
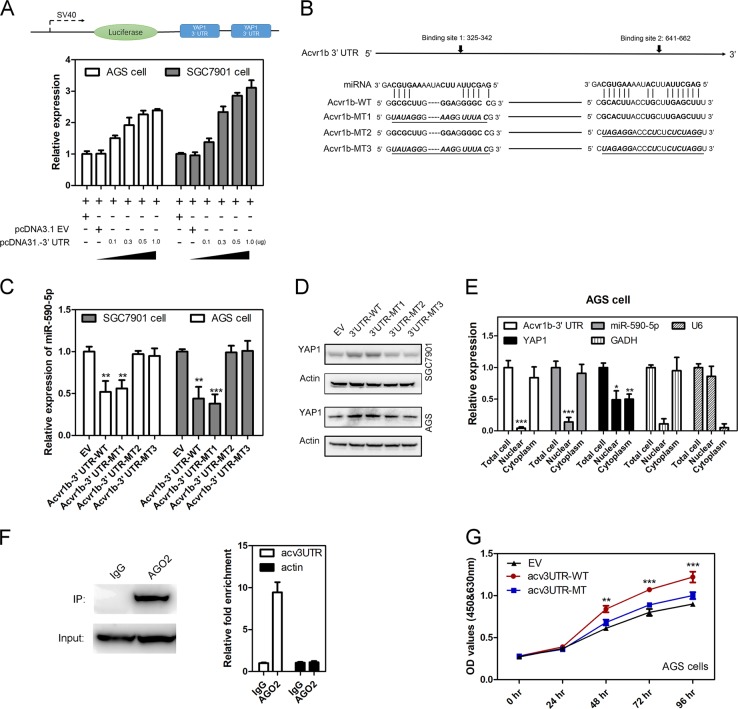
Acv3UTR-active YAP1 expression by absorbing miR-590-5p. **a** Luciferase reporter assays. AGS or SGC7901 cells were cotransfected with the miR-590-5p sensor construct and pcDNA3.1-empty vector (EV) or different doses of pcDNA3.1-3ʹUTR. **b** Predicted biding sites of miR-590-5p in *acv*3UTR and mutant strategy. **c** MiR-590-5p expression level based on empty vector (EV), wild type 3ʹUTR, binding site 1 mutant type (MT1), binding site 2 mutant type (MT2) or dual sites mutant type (M3). **d** YAP1 level based on empty vector (EV), wild type 3ʹUTR, binding site 1 mutant type (MT1), binding site 2 mutant type (MT2) or dual sites mutant type (M3). **e** Cell localization of Acv3UTR, miR-590-5p, and YAP1 in AGS cells. **f** RIP was carried out using anti-Ago2 antibody in extraction of AGS cells. YAP1 (upper) and acv3UTR (lower) were enriched. **g** Mutation of *Acv*3UTR miR-590-5p binding site abolished promotion effects on AGS cells proliferation. *P < 0.05; **P < 0.01; ***P < 0.001 (paired *t*-test, two tailed).

To further demonstrate *acv*3UTR act as a sponge to sequester miR-590-5p, we performed luciferase reporter assay using YAP1 luciferase construct (Fig. 5a, upper). As a result, the repression effect of miR-590-5p on the luciferase activity of YAP1 luciferase construct was ameliorated by the cotransfection of increasing amounts of *acv*3UTR (Fig. 5a, lower). There are two predicted binding sites of miR-590-5p in *acv*3UTR (Fig. 5b). We constructed different overexpression plasmids containing fragments, including wide type (Acvr1b-UTR-WT), single-site-mutated types Acvr1b-UTR-MT1 and Acvr1b 3ʹUTR-MT2, and dual-site mutated type Acvr1b-UTR-MT3 (Fig. 5b). After 48 h transfection, miR-590-5p was lowered by Acvr1b-UTR-WT transfection (Fig. 5c). Acvr1b-UTR-MT1 showed the same miR-590-5p level as Acvr1b-UTR-WT, while Acvr1b-UTR-MT2 and dual-site mutated type (Acvr1b-UTR-MT3) abolished the inhibition effect to miR-590-5p, indicating that site 2 was the true binding site of miR-590-5p. Besides, YAP1 exhibited higher level in Acvr1b-UTR-WT transfected cells and Acvr1b-UTR-MT1 transfected cells while no change was found in Acvr1b-UTR-MT2 transfected and dual-site mutated type transfected cells (Fig. 5d).

Then, nuclear and cytoplasmic fractions subcellular location of acv3UTR, miR-590-5p, and YAP1 were detected. GAPDH and U6 served as cytoplasmic and nuclear localization controls, respectively. As a result, *acv*3UTR and miR-590-5p were mainly distributed in the cytoplasm, while YAP1 was distributed in both the nucleus and cytoplasm (Figs. 5e and S2A).

To test whether *acv*3UTR and YAP1 associate with Ago2, the core component of the RNA-induced silencing complex, associates with miRNAs to form miRNA ribonucleoprotein complexes [36, 37]; RNA immunoprecipitation (RIP) was carried out using an antibody against Ago2 in extraction of AGS cells. As a result, YAP1 and *acv*3UTR were preferentially enriched in Ago2-containing miRNPs relative to control IgG (Fig. 5f). Moreover, the enhancing effect of *acv*3UTR on GC cell proliferation was abolished by miR-590-5p binding site mutant (Figs. 5g and S2B). Clinic expression correlation study of acvr1B, miR-590, and YAP1 showed that *acv*3UTR expression was negatively relevant to miR-590-5p expression and positively relevant to YAP1 expression (Fig. S2C).

Taken together, these results strongly suggest that *acv*3UTR physically interacts with miR-590-5p, and regulates its expression and activity, which promotes GC cell proliferation.

### CFIm25 contributed to *acv*3UTR aberrant expression in GC

**Fig. 6:**
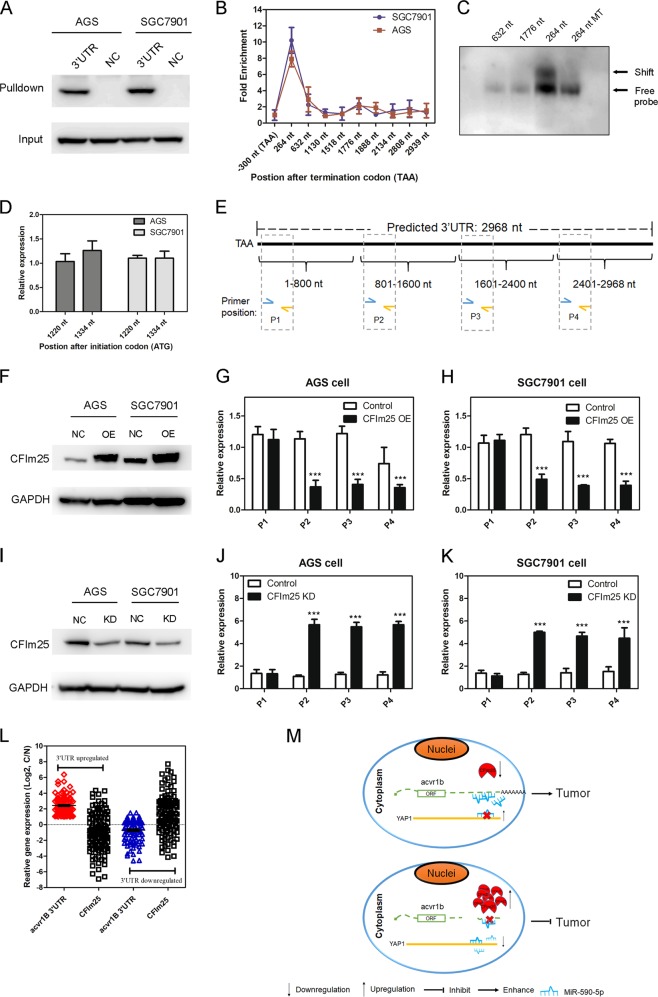
CFIm25 is the key regulator of Acv3UTR level in GC cells. **a** CFIm25 was enriched by Acv3UTR-pulldown assay. **b** RIP-PCR demonstrated that the UGUA-1518 motif (UGUA site 1518 nt after termination codon TAA) is the binding site of CFIm25. Enrichment fold was normalized to input. **c** EMSA demonstrated the interaction between CFIm25 and the fragment containing UGUA site 1518. **d** CFIm25 did not bind to UGUA sites in ORF region. **e** Strategy of primer design for detection of different fragment in Acv3UTR. **f**–**h** CFIm25 overexpression lowered the level of fragments contained after verification of the UGUA motif. **i**–**k** CFIm25 knockdown enhanced the level of fragments containing or after verified UGUA motif. **l** *Acv*3UTR and CFIm25 expression were validated in 224 paired GC samples with qRT-PCR. Cutoff=2. **m** Model depicting the proposed oncogenic action of the *acv*3UTR. ***P < 0.001 (paired *t*-test, two tailed).

To explore the mechanism causing upregulation of acv3UTR, we chose several master 3′UTR regulators for investigation, which have previously been demonstrated to bind 3ʹUTR promoting their decay or increasing their stability, including CFIm25 [22], AUF1 [38], HuR [39], TTP [40], and KSRP [41] RNA pulldown assay demonstrated that CFIm25 was enriched by *acv*3UTR (Fig. 6a). CFIm25 was reported to specifically bind the poly(A) site upstream element UGUA [20]. Then we designed primers for different region containing UGUA motif on 3ʹUTR and RIP-qPCR was performed using CFIm25 antibody, demonstrated that the UGUA motif at 264 nt after termination codon was significantly enriched by CFIm25 (Fig. 6b). The sequences at 1130 and 1176 nt were also enriched but not significant. We thought this may be due to some fragments containing 1518 nt UGUA motif also covered 1130 nt and 1176 nt since they were very closed in sequence. EMSA further verified that only the UGUA motif at 1518 nt was the real CFIm25-binding site (Fig. 6c). We also demonstrated that the two UGUA motifs in Acvr1B ORF region were not in the CFIm25-binding site (Fig. 6d). We next designed qRT-PCR primers for specific region (Fig. 6d). After CFIm25 overexpression or knockdown, only the fragments containing or after verified UGUA motif were changed accordingly (Fig. 6f–k).

Importantly, CFIm25 was downregulated in GC tissue, the expression of which was frequently reverse to the expression of *acv*3UTR in GC patients (Figs. 6l and S3A). CFIm25 overexpression leaded to suppression of GC cell proliferation while knocking promoted proliferation (Fig. S3B–E). Kaplan–Meier survival analysis showed that the overall survival rates among patients with GC were significantly higher in patients with high CFIm25 level than in those with low CFIm25 level (Fig. S3F). These results suggested that CFIm25 contributed to *acv*3UTR aberrant expression in GC, and plays a tumor-suppressor role.

In summary, 3ʹUTR of acvr1B (*acv*3UTR) played roles as an oncogenic lncRNA and accelerated GC progression through miR-590-5p/YAP1 axis. Expression of *acv*3UTR was controlled by CFIm25. In normal gastric tissue, CFIm25 was highly expressed, leading to shortening of 3ʹUTR of acvr1B. The reduced expression of *acv*3UTR leaded to the loss of sponging effect of *acv*3UTR to miR-590-5p, the releasing of which inhibited its targeted oncogene YAP1. While in GC tissue, CFIm25 was downregulated, leading to the increasing amount of *acv*3UTR in cells. The increased *acv*3UTR competingly sponging miR-590-5p reduced the suppressing effect of miR-590-5p to YAP1 (Fig. 6m).

## Discussion

We described a 3ʹUTR from coding gene acvr1B functioned as an oncogenic noncoding RNA in GC. From previous studies, acvr1B might take part in tumorigenesis but the function of which was still controversial. For instance, homozygous deletion of acvr1b is associated with an aggressive cancer phenotype in pancreatic cancer [27]. But there is also research demonstrated that acvr1b suppressed early stages of pancreatic tumorigenesis [28]. In small-bowel adenocarcinoma, exome-wide somatic mutation characterization identified acvr1b and acvr2a as novel candidate driver genes [29]. In prostate cancer cells acvr1b promoted lymph node metastasis [30]. Acvr1b knockdown results in inhibition of monocyte/macrophage differentiation in leukemia [31]. To our knowledge, there is no study about the function of acvr1B in GC. Our data suggested Acvr1B protein might not have regulatory function but its 3ʹUTR worked as an oncogene in GC.

There are researches highlighted that alteration of 3ʹUTRs could regulate the malignant phenotype of cancer cells [17]. The 3ʹUTR has been mostly implicated in ceRNA regulation in *trans* in many types of cancer [42]. For instance, in lung cancer, PTCH1-3ʹUTR promotes cell metastasis by absorbing miR-101-3p [43]. In breast cancer, CYP4Z1 3ʹUTR represses migration and EMT by sponging miR-9 [44]. In osteosarcoma, IGF1 3ʹUTR promotes angiogenesis by sponging miR-29 family [45].

There are five miRNAs mostly enriched by *acv*3UTR pulldown. MiR-199a-5p has been shown to interact with acvr1B during monocyte/macrophage differentiation [31, 46], indicating our screening system worked well for screening of miRNAs interacting with acv3UTR. Mizuno et al. demonstrated that the acvr1B gene was a target for miR-210 through which miR-210 promoted osteoblastic differentiation [47]. However, miR-210 was not enriched by *acv*3UTR in GC, suggesting that the miRNA sponging ability of *acv*3UTR could be cell types dependent. MiR-590-5p is the mostly enriched miRNA among these pulled down miRNAs. Previous research showed that it functioned as a tumor suppressor or an oncogene based on cancer type. For example, in renal carcinoma cells, miR-590-5p enhanced cancer cell proliferation [48]. While in hepatocellular carcinoma and colorectal cancer miR-590-5p plays tumor suppressor roles [35, 49]. There is a recently published paper in GC delineating that miR-590-5p functions as a tumor suppressor by targeting FGF18 [50]. In our studies, we found the same function of miR-590-5p in GC. Compared with their studies, we demonstrated how miR-590-5p was regulated in GC. In fact, when overexpression acv3UTR in GC cell lines, FGF18 expression was also enhanced (Fig. S4). These results indicate that acv3UTR/miR-590-5p axis could have global regulatory effects in GC by regulating miR-590-5p target genes.

Here, we demonstrated miR-590-5p as a tumor suppressor in GC by negatively regulating YAP1, a major downstream effector of the Hippo pathway. YAP is a transcriptional activator pervasively induced in several types of human cancers [51]. In GC, both YAP mRNA and protein levels are found frequently upregulated, and elevated YAP protein expression and nuclear localization correlate with bad patients’ outcome [52, 53]. YAP1 had first been thought of as a nuclear effector [54]. However, more and more evidences showed that it located in both nucleus and cytoplasm [55, 56]. Some factors such as phosphorylation and cell density can regulate its subcellular localization [55, 57]. After analysis of subcellular location, we confirmed that *acv*3UTR, miR-590-5p and YAP1 could be colocalized in cytoplasm which provided the possibility of interaction. YAP has an evolutionarily conserved paralog named TAZ. In this study, we detected the TAZ level after overexpression of acv3UTR, but found no significant change (Fig. S5A). We also cloned four TAZ fragments containing potential miR-590-5p binding sites and performed dual-luciferase assay, demonstrated that there was no significant binding of miR-590-5p to TAZ (Figs. S5B and S5C). These results indicate that acv3UTR regulates YAP1 protein but not TAZ even though there is more than 40% amino acid identity between human TAZ and human YAP [58]. However, our current results cannot explain how miR-590-5p selectively targeting YAP1 but not TAZ since they share similar sequences. Further studies need to be done.

Finally, we investigated the mechanism that leads to aberrant *acv*3UTR expression in GC. We demonstrated CFIm25, a 3′UTR shortening regulator, contributed to *acv*3UTR aberrant expression in GC. In glioblastoma, CFIm25 was identified as a broad repressor of proximal poly(A) site usage and deletion of which increases cell proliferation [22]. Our data suggested that the loss of CFIm25 in GC contributes to the disruption of *acv*3UTR degradation, which further led to aberrant regulation of miR-590-5p/YAP1 axis. Since previous study showed that CFIm25 had a global function in regulation of 3ʹUTR, it might also regulate the stability of 3ʹUTR of a subset genes besides acvr1B. Hence, RNA sequencing from the CFIm25 wild-type and knockout GC cell will be helpful for identifying CFIm25-regulated 3ʹUTR in the future.

In conclusion, we elucidated that CFIm25 regulated *acv*3UTR can function as an ceRNA and stimulate GC tumorigenesis via modulating the miR-590-5p/YAP1 axis. Our findings provide a novel insight into the potential mechanism of the pathogenesis of *acv*3UTR in GC.

## Materials and methods

Mainly materials and methods were described as below. More detail can be found in supplementary Materials and methods.

### Patients and tissue samples

Pairs of matched GC samples and adjacent normal counterparts were collected from 224 patients (121 males and 103 females). We collected the tissue samples on condition of receiving the approval of Clinical Research Ethics Committee as well as obtaining written informed consent from all participants. All methods were performed in accordance with the relevant guidelines and regulations of Clinical Research Ethics Committee. After surgical resection, tissue samples were immediately frozen and stored in liquid nitrogen until RNA or protein extraction.

### Cell culture and transfection

Human GC cell lines AGS and SGC7901 were purchased from the Cancer Institute of the Chinese Academy of Medical Sciences, and maintained in RPMI-1640 medium in regular conditions. The cells were transfected using Lipofactamine3000 reagent (Life, USA).

### RNA extraction, cDNA synthesis, and RT-PCR assays

We used Trizol reagent (Life, USA) for extracting of total RNA. SuperscriptIII reverse transcriptase (Invitrogen, USA) was used for single-strand cDNA synthesis. Oligo (dT)_18_ RT primers and random primers (TaKaRa, China) were used for mRNAs and lncRNAs. A stem loop RT primer were respectively used for the reverse transcription of miRNAs. SYBR mix kit (TaKaRa, China) was used for mRNA and lncRNA detection. TaqMan MicroRNA Assays (Applied Biosystems) was used for miRNA detection. The relative expression amounts were measured using the 2^–ΔΔCT^ method.

### Nuclear–cytoplasmic fractionation

Nuclear/cytoplasmic fractionation was conducted by the Protein and RNA Isolation System (Thermo Fisher, USA). U6 was treated as a nuclear control, while GAPDH was a cytoplasmic control.

### Cell proliferation, migration, and invasion analysis

GC cell proliferation was detected by CCK8 reagent (DOJINDO, Japan). Experiments were repeated three times in five replicates. The wound healing assay was used to evaluate cell migration. Cell invasion was assessed using BioCoat Matrigel Invasion Chamber (BD Biosciences, USA).

### Dual-luciferase reporter assay

The luciferase activity was assessed by Dual-Luciferase Reporter Assay System (Promega, USA), and the firefly luciferase activity was normalized by renilla luciferase activity. All the experiments were repeated three times in five replicates.

### In vivo nude mice tumor formation studies

The IACUC committee of Shandong University approved all animal experiments. Animal maintenance and experimental procedures were performed following strict adherence to the guidelines of the Animal Care and Use Committee. A total of 1 × 10^7^ cells in 80 μl of PBS were subcutaneously transplanted into 6-week-old BALB/c female nude mice. Tumor volumes were measured every 3 days and calculated based on the equation: *V* = (length × width [2])/2. Five mice were used for each group.

### Western blotting, histology experiments, and HE staining

Western blotting was performed according to the standard protocol with the following antibody dilutions: Anti-acvr1b (Abcam, ab109300): 1:1000; Anti-YAP1 (Abcam, ab52771): 1:1500; Anti-CFIm25 (Proteintech, 10322-1-AP): 1:2000. Anti-Actin (Proteintech, 23660-1-AP): 1:5000. Anti-GAPDH (Proteintech, 60004-1-Ig): 1:30000. ECL reagent was used for imaging (Millipore, USA). Histology experiments and HE staining were performed using histology experiment standard staining kit (ZhongshanJinqiao, China).

### RNA immunoprecipitation (RIP)

RIP assay was conducted with a Magna RNA-binding protein immunoprecipitation kit (Thermo Fisher Scientific, USA). Mouse IgG was used as negative control. For RIP-PCR, enrichment was normalized to input.

### Electrophoretic mobility shift assay (EMSA)

EMSA was performed using LightShift Chemiluminescent EMSA Kit (Thermo Fisher, USA) according to previous publications [59].

### RNA–protein interaction assay

We performed RNA-pulldown assay to detect the RNA–protein interaction using Thermo Scientific Pierce Magnetic RNA-Protein Pull-Down Kit (Thermo Fisher Scientific, USA) according to the paper and previous research [60].

### Statistics

Each experiment was repeated at least three times. A two-tailed student’s *t*-test was performed. *p < 0.05; **p < 0.01; ***p < 0.001. The mean ± SD is displayed in the figures.

## Supplementary information

Supplemental Figure 1

Supplemental Figure 2

Supplemental Figure 3

Supplemental Figure 4

Supplemental Figure 5

Supplemental Figure legends

Supplemental table 1

Supplemental table 2

Supplemental methods

Supplemental sequence
