# Supplementary figures and images for: CFIm25-regulated lncRNA acv3UTR promotes gastric tumorigenesis via miR-590-5p/YAP1 axis
Source: Oncogene. 2020 Feb 17;39(15):3075–88. doi: 10.1038/s41388-020-1213-8 (PMC7142022; doi:10.1038/s41388-020-1213-8)

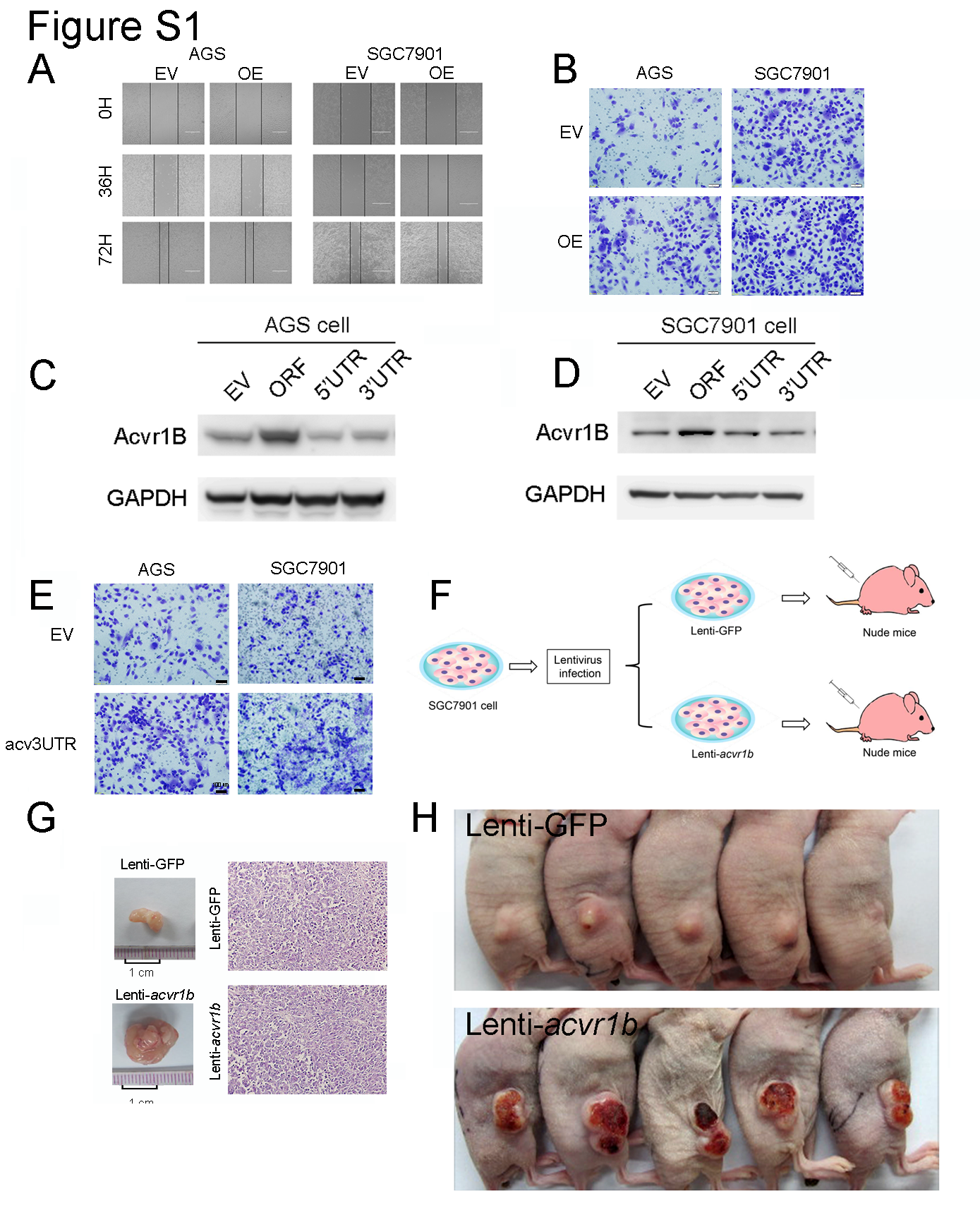

Supplement: Supplementary file 1 — Supplemental Figure 1 [file 41388_2020_1213_MOESM1_ESM.tif]

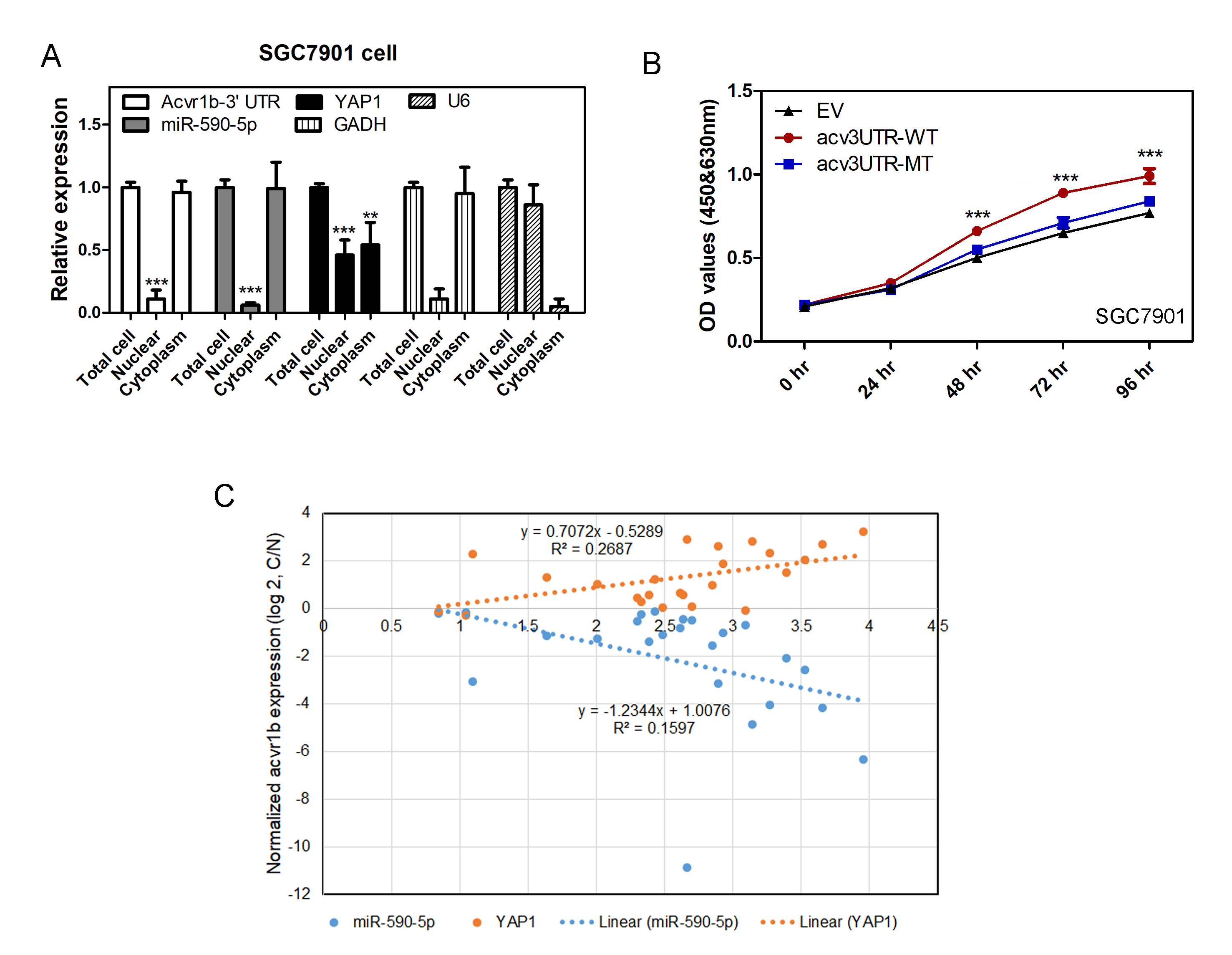

Supplement: Supplementary file 2 — Supplemental Figure 2 [file 41388_2020_1213_MOESM2_ESM.tif]

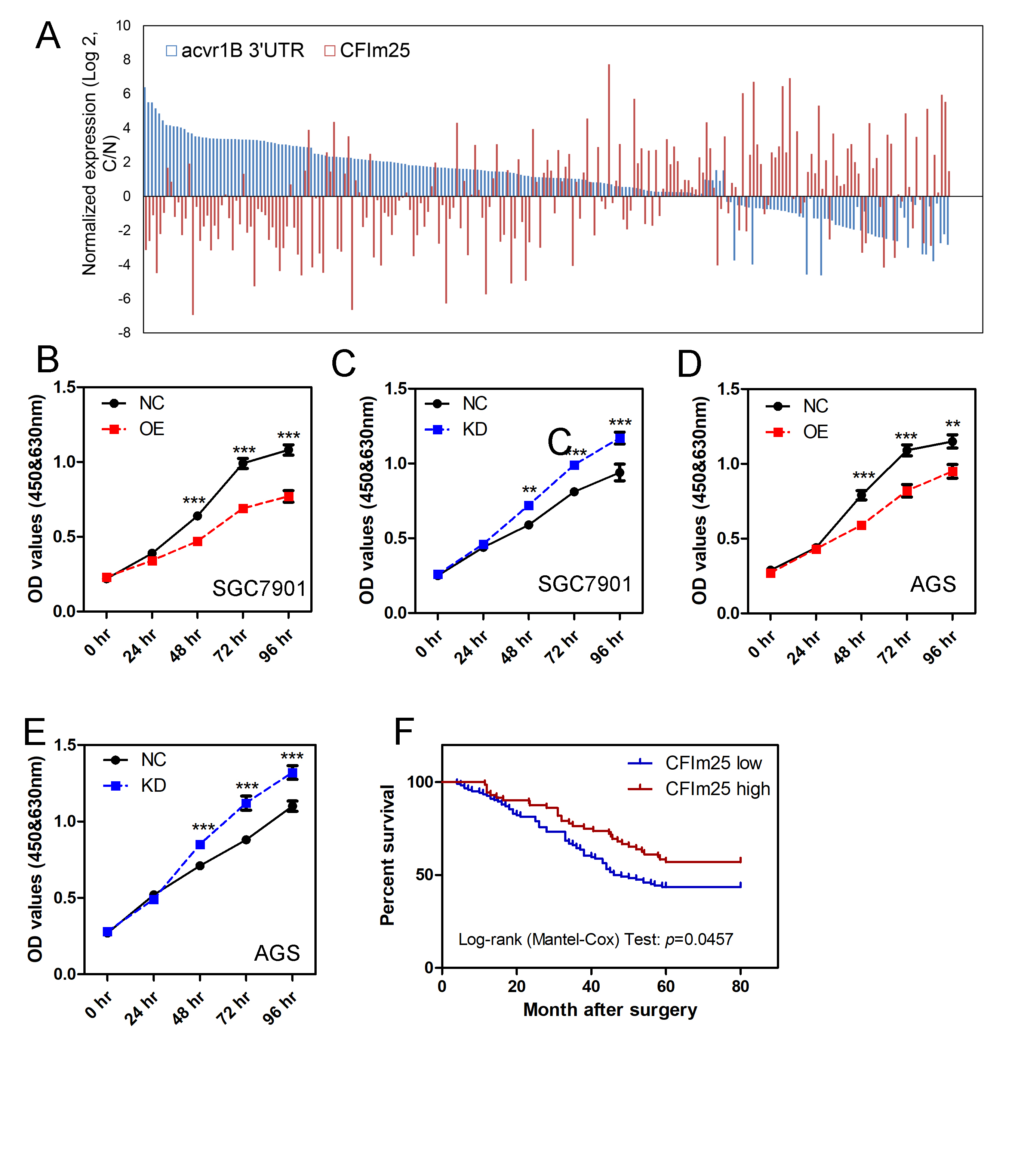

Supplement: Supplementary file 3 — Supplemental Figure 3 [file 41388_2020_1213_MOESM3_ESM.tif]

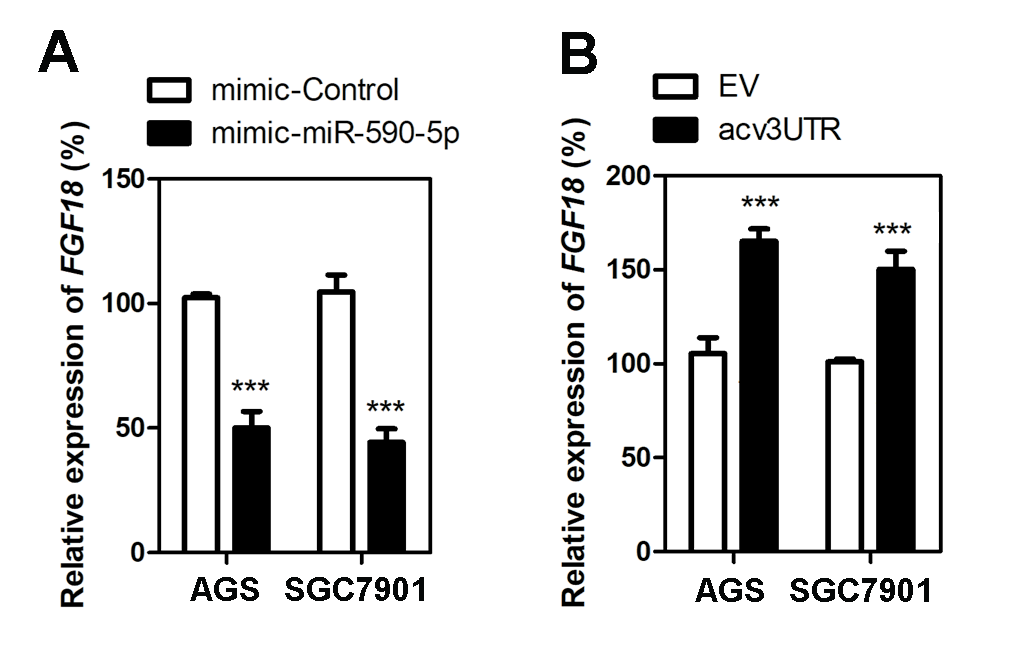

Supplement: Supplementary file 4 — Supplemental Figure 4 [file 41388_2020_1213_MOESM4_ESM.tif]

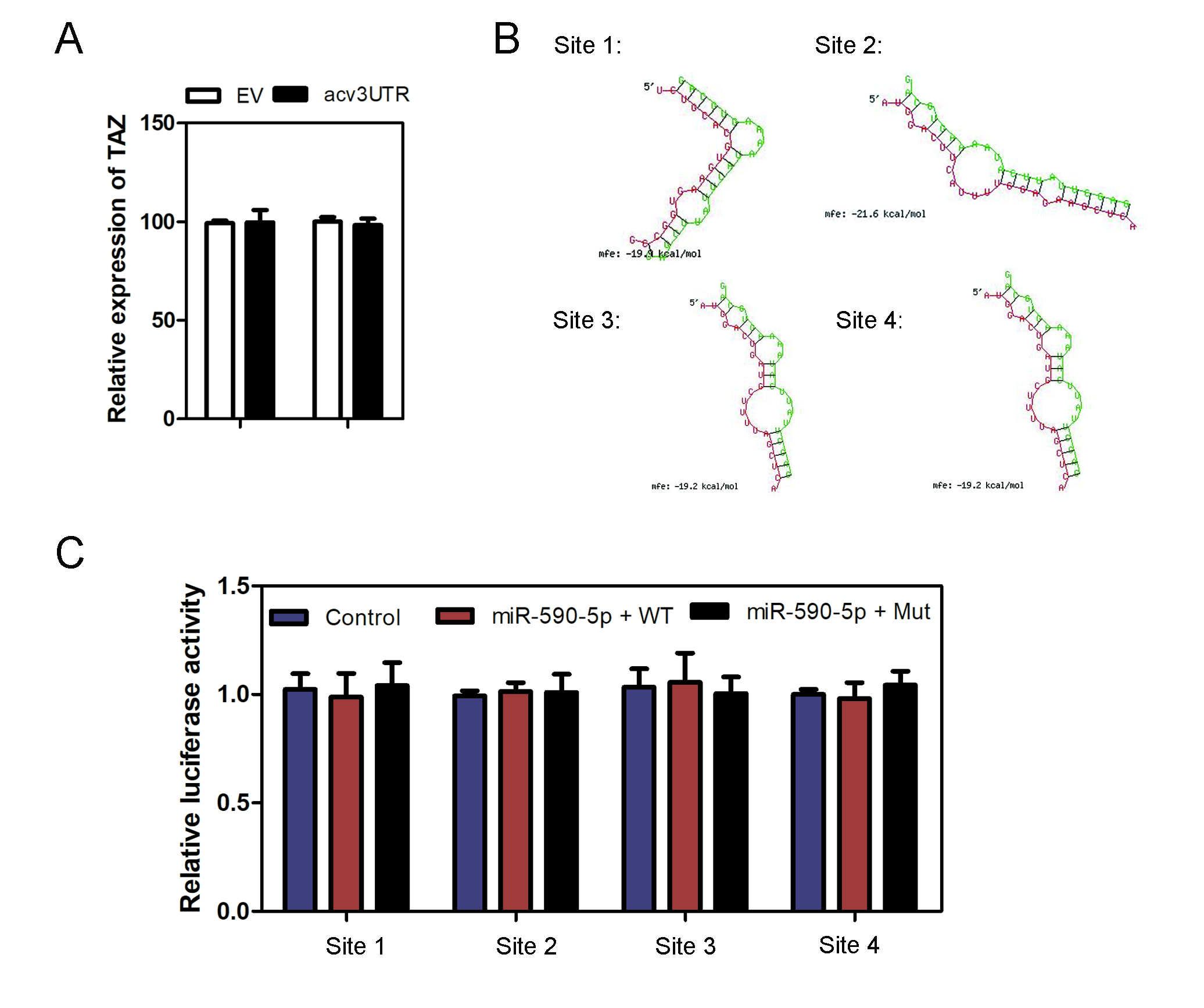

Supplement: Supplementary file 5 — Supplemental Figure 5 [file 41388_2020_1213_MOESM5_ESM.tif]
