# Supplementary material for: CFIm25-regulated lncRNA acv3UTR promotes gastric tumorigenesis via miR-590-5p/YAP1 axis
Source: Oncogene. 2020 Feb 17;39(15):3075–88. doi: 10.1038/s41388-020-1213-8 (PMC7142022; doi:10.1038/s41388-020-1213-8)
Supplement: Supplementary file 6 — Supplemental Figure legends [file 41388_2020_1213_MOESM6_ESM.docx]

**Supplementary figure legend.**

**Figure S1 *Acv*3UTR is an oncogene. A** Representative photos of migration of AGS cell and SGC7901 cell transfected with negative control vector (EV) or full length acvr1b overexpression plasmid (OE). **B** Representative photos of invasion of AGS cell and SGC7901 cell transfected with negative control vector (EV) or full length acvr1b overexpression plasmid (OE). **C** Protein level of Acvr1b after transfection of ORF, 5’ UTR or 3’UTR in AGS cells. **D** Protein level of Acvr1b after transfection of ORF, 5’ UTR or 3’UTR in SGC7901 cells. **E** Representative photos of GC cell invasion assay with negative control vector (EV) or *acv*3UTR overexpression vector. **F** In vivo xenograft node mice model experiment strategy. Negative control: Lenti-GFP. *Acv*3UTR stable overexpression: Lenti-acvr1b. **G** Representative photo of tumors and H&E staining. **H** Raw photos of all tumors on node mice.

**Figure S2 Acv3UTR active YAP1 expression by absorbing miR-590-5p. A** Cell localization of *acv*3UTR, miR-590-5p and YAP1 in SGC7901 cells. **B** Mutation of Acv3UTR miR-590-5p binding site abolished promotion effects on SGC7901 cells proliferation. **C** Clinic expression correlation study of acvr1B, miR-590, and YAP1.

**Figure S3 Tumor suppressor role of CFIm25 in GC. A** CFIm25 showed reverse expression pattern to *acv*3UTR in GC patients (n=224). C: GC tissues. N: GC adjacent tissues. **B-D** CFIm25 overexpression leaded to suppression of GC cell proliferation while knocking promoted proliferation. NC: negative control. OE: CFIm25 overexpression. KD: CFIm25 knockdown. **F** Overall survival rates among patients with GC were significantly higher in patients with high CFIm25 level than in those with low CFIm25 level.

**Figure S4 *Acv*3UTR might enhance FGF18 level. A** Relative FGF18 expression with or without miR-590-5p overexpression. **B** Relative FGF18 expression with or without *acv*3UTR overexpression. NC: negative control.

**Figure S5 Neither acv3UTR nor miR-590-5p affects TAZ level. A** Relative TAZ expression with or without *acv*3UTR overexpression. NC: negative control. B Potential binding sites of miR-590-5p on TAZ. **C** Relative luciferase activity with or without miR-590-5p overexpression.
