# Supplementary material for: CFIm25-regulated lncRNA acv3UTR promotes gastric tumorigenesis via miR-590-5p/YAP1 axis
Source: Oncogene. 2020 Feb 17;39(15):3075–88. doi: 10.1038/s41388-020-1213-8 (PMC7142022; doi:10.1038/s41388-020-1213-8)
Supplement: Supplementary file 7 — Supplemental table 1 [file 41388_2020_1213_MOESM7_ESM.docx]

**Table S1 Comparison of acvr1b expression levels with clinic-pathological features in patients with primary gastric cancer**

| **Clinic-pathological parameters** | | **N** | **Mean ± SD**^1^ | ***P*** |
| --- | --- | --- | --- | --- |
| **Age, y**  ≥60  <60  **Gender**  Male  Female  **Vascular invasion**^2,3^  Negative  Positive  **Perineural invasion**^2,3^  Negative  Positive  **Location**^2,3^  Proximal  Body  Distal  **Lauren's Classification**^2,3^  Intestinal  Diffuse  Mixed  **Grade**  Differentiated  poorly differentiated  **Tumor size**  T2  T3  T4  **Lymphayic invasion**^2,3^  N0  N1  N2  N3  **Nodal involvement**  M0  M1  **TNM Stage**^2,3^  Stage 1+2  Stage 3+4  ***Helicobacter pylori***  Positive  Negative | 117  107  89  135  104  120  99  125  76  66  82  79  83  62  135  89  86  95  43    58  73  48  45    121  103  110  114  86  138 | | 1.5588±0.20688  1.631±0.24188  1.61±0.17533  1.7211±0.36178  1.3058±0.22201  2.4706±0.22276  1.2042±0.2209  1.1994±0.24297  1.4593±0.23023  1.2251±0.38408  0.9123±0.25029  1.2935±0.18954  0.8341±0.40568  1.2066±0.41634  1.2643±0.25035  1.2435±0.23267  0.4889±0.48093  1.2617±0.30298  1.9799±0.19605  1.5481±0.40534  1.0378±0.20643  1.3948±0.40522  1.1843±0.49279  1.1721±0.16378  1.7358±0.59616  0.9005±0.80223  1.1104±0.31235  1.3009±0.19943  2.3419±0.53338 | 0.098  0.171  0.026  0.947  0.295  0.59  0.953  0.022  0.649  0.275  0.0426  0.0418 |

1. Mean ± SD, mean of Log_1.5_ (C/N), Standard Definition of Log2 (C/N). C, normalized expression of cancer tissues; N, normalized expression of adjacent noncancerous tissues.
2. The definitions of location, stages and the criteria for histological classification followed the World Health Organization classification and the Japanese classification for GC.
3. Major reference of Clinic-pathological parameters: Vascular invasion:^1^ . Perineural invasion: ^2^. Location: ^3^. Lauren's Classification:^4^. TNM stage:^5^.

1 Gresta LT, Rodrigues-Junior IA, de Castro LP, Cassali GD, Cabral MM. Assessment of vascular invasion in gastric cancer: a comparative study. World J Gastroenterol 2013; 19: 3761-3769.

2 Tanaka A, Watanabe T, Okuno K, Yasutomi M. Perineural invasion as a predictor of recurrence of gastric cancer. Cancer 1994; 73: 550-555.

3 Dicken BJ, Bigam DL, Cass C, Mackey JR, Joy AA, Hamilton SM. Gastric adenocarcinoma: review and considerations for future directions. Ann Surg 2005; 241: 27-39.

4 Lauren P. The Two Histological Main Types of Gastric Carcinoma: Diffuse and So-Called Intestinal-Type Carcinoma. An Attempt at a Histo-Clinical Classification. Acta Pathol Microbiol Scand 1965; 64: 31-49.

5 Tokunaga H, Shimada M, Ishikawa M, Yaegashi N. TNM classification of gynaecological malignant tumours, eighth edition: changes between the seventh and eighth editions. Jpn J Clin Oncol 2019; 49: 311-320.
