# Supplementary material for: CFIm25-regulated lncRNA acv3UTR promotes gastric tumorigenesis via miR-590-5p/YAP1 axis
Source: Oncogene. 2020 Feb 17;39(15):3075–88. doi: 10.1038/s41388-020-1213-8 (PMC7142022; doi:10.1038/s41388-020-1213-8)
Supplement: Supplementary file 9 — Supplemental methods [file 41388_2020_1213_MOESM9_ESM.docx]

**Supplementary Materials and methods**

1. **Vascular invasion, Lymphayic invasion, Perineural invasion, Location and Lauren's Classification.** The definitions of location, stages and the criteria for histological classification followed the World Health Organization classification^1^ and the Japanese classification for GC ^2^. All surgical specimens of the primary tumors and regional lymph nodes had been processed and examined histologically by routine HE staining. The resected primary tumors and regional lymph nodes were reviewed histologically by two pathologists using HE staining.
2. **Prediction of miRNA-RNA interaction.** MiRNAs’ binding sites, potential acv3UTR binding miRNAs were predicted by Targetscan^3^ . Binding sites of miR-590-5p on TAZ were predicted by RNAhybrid^4^

**Reference**

1 Aaltonen LA HS, World Health Organization, International Agency for Research on Cancer. Pathology and genetics of tumours of the digestive system. Lyon Oxford: IARC Press/Oxford University Press distributor; 2000.

2 Japanese Gastric Cancer A. Japanese classification of gastric carcinoma: 3rd English edition. Gastric Cancer 2011; 14: 101-112.

3 Agarwal V, Bell GW, Nam JW, Bartel DP. Predicting effective microRNA target sites in mammalian mRNAs. Elife 2015; 4.

4 Rehmsmeier M, Steffen P, Hochsmann M, Giegerich R. Fast and effective prediction of microRNA/target duplexes. RNA 2004; 10: 1507-1517.
