# Supplementary material for: CFIm25-regulated lncRNA acv3UTR promotes gastric tumorigenesis via miR-590-5p/YAP1 axis
Source: Oncogene. 2020 Feb 17;39(15):3075–88. doi: 10.1038/s41388-020-1213-8 (PMC7142022; doi:10.1038/s41388-020-1213-8)
Supplement: Supplementary file 10 — Supplemental sequence [file 41388_2020_1213_MOESM10_ESM.docx]

# Supplementary data: sequence information

# ACVR1B 5’UTR

# (Homo sapiens activin A receptor type 1B (ACVR1B), transcript variant 2, mRNA. NCBI Reference Sequence: NM_020327.3)

# AATGCTTCTCCTAAGCACCTCGTGTGTGTTCTTCGGCCTCACTGCTCTGTGGCTTAGGTATCTGTGCTGTGGGGTTTGAGAAACATGGTGAAGGTGTATGAACAGAGCTTGACATTTGTGCTCTGCTGTGTGCGTGCACCAGCTGCCTCCAGGCCAACTACACGTGTGAGACAGATGGGGCCTGC

# ACVR1B ORF

# (Homo sapiens activin A receptor type 1B (ACVR1B), transcript variant 1, mRNA. NCBI Reference Sequence: NM_004302.4)

ATGGCGGAGTCGGCCGGAGCCTCCTCCTTCTTCCCCCTTGTTGTCCTCCTGCTCGCCGGCAGCGGCGGGTCCGGGCCCCGGGGGGTCCAGGCTCTGCTGTGTGCGTGCACCAGCTGCCTCCAGGCCAACTACACGTGTGAGACAGATGGGGCCTGCATGGTTTCCATTTTCAATCTGGATGGGATGGAGCACCATGTGCGCACCTGCATCCCCAAAGTGGAGCTGGTCCCTGCCGGGAAGCCCTTCTACTGCCTGAGCTCGGAGGACCTGCGCAACACCCACTGCTGCTACACTGACTACTGCAACAGGATCGACTTGAGGGTGCCCAGTGGTCACCTCAAGGAGCCTGAGCACCCGTCCATGTGGGGCCCGGTGGAGCTGGTAGGCATCATCGCCGGCCCGGTGTTCCTCCTGTTCCTCATCATCATCATTGTTTTCCTTGTCATTAACTATCATCAGCGTGTCTATCACAACCGCCAGAGACTGGACATGGAAGATCCCTCATGTGAGATGTGTCTCTCCAAAGACAAGACGCTCCAGGATCTTGTCTACGATCTCTCCACCTCAGGGTCTGGCTCAGGGTTACCCCTCTTTGTCCAGCGCACAGTGGCCCGAACCATCGTTTTACAAGAGATTATTGGCAAGGGTCGGTTTGGGGAAGTATGGCGGGGCCGCTGGAGGGGTGGTGATGTGGCTGTGAAAATATTCTCTTCTCGTGAAGAACGGTCTTGGTTCAGGGAAGCAGAGATATACCAGACGGTCATGCTGCGCCATGAAAACATCCTTGGATTTATTGCTGCTGACAATAAAGATAATGGCACCTGGACACAGCTGTGGCTTGTTTCTGACTATCATGAGCACGGGTCCCTGTTTGATTATCTGAACCGGTACACAGTGACAATTGAGGGGATGATTAAGCTGGCCTTGTCTGCTGCTAGTGGGCTGGCACACCTGCACATGGAGATCGTGGGCACCCAAGGGAAGCCTGGAATTGCTCATCGAGACTTAAAGTCAAAGAACATTCTGGTGAAGAAAAATGGCATGTGTGCCATAGCAGACCTGGGCCTGGCTGTCCGTCATGATGCAGTCACTGACACCATTGACATTGCCCCGAATCAGAGGGTGGGGACCAAACGATACATGGCCCCTGAAGTACTTGATGAAACCATTAATATGAAACACTTTGACTCCTTTAAATGTGCTGATATTTATGCCCTCGGGCTTGTATATTGGGAGATTGCTCGAAGATGCAATTCTGGAGGAGTCCATGAAGAATATCAGCTGCCATATTACGACTTAGTGCCCTCTGACCCTTCCATTGAGGAAATGCGAAAGGTTGTATGTGATCAGAAGCTGCGTCCCAACATCCCCAACTGGTGGCAGAGTTATGAGGCACTGCGGGTGATGGGGAAGATGATGCGAGAGTGTTGGTATGCCAACGGCGCAGCCCGCCTGACGGCCCTGCGCATCAAGAAGACCCTCTCCCAGCTCAGCGTGCAGGAAGACGTGAAGATCTAA

**ACVR1B 3’UTR**

# (Homo sapiens activin A receptor type 1B (ACVR1B), transcript variant 1, mRNA. NCBI Reference Sequence: NM_004302.4)

CTGCTCCCTCTCTCCACACGGAGCTCCTGGCAGCGAGAACTACGCACAGCTGCCGCGTTGAGCGTACGATGGAGGCCTACCTCTCGTTTCTGCCCAGCCCTCTGTGGCCAGGAGCCCTGGCCCGCAAGAGGGACAGAGCCCGGGAGAGACTCGCTCACTCCCATGTTGGGTTTGAGACAGACACCTTTTCTATTTACCTCCTAATGGCATGGAGACTCTGAGAGCGAATTGTGTGGAGAACTCAGTGCCACACCTCGAACTGGTTGTAGTGGGAAGTCCCGCGAAACCCGGTGCATCTGGCACGTGGCCAGGAGCCATGACAGGGGCGCTTGGGAGGGGCCGGAGGAACCGAGGTGTTGCCAGTGCTAAGCTGCCCTGAGGGTTTCCTTCGGGGACCAGCCCACAGCACACCAAGGTGGCCCGGAAGAACCAGAAGTGCAGCCCCTCTCACAGGCAGCTCTGAGCCGCGCTTTCCCCTCCTCCCTGGGATGGACGCTGCCGGGAGACTGCCAGTGGAGACGGAATCTGCCGCTTTGTCTGTCCAGCCGTGTGTGCATGTGCCGAGGTGCGTCCCCCGTTGTGCCTGGTTCGTGCCATGCCCTTACACGTGCGTGTGAGTGTGTGTGTGTGTCTGTAGGTGCGCACTTACCTGCTTGAGCTTTCTGTGCATGTGCAGGTCGGGGGTGTGGTCGTCATGCTGTCCGTGCTTGCTGGTGCCTCTTTTCAGTAGTGAGCAGCATCTAGTTTCCCTGGTGCCCTTCCCTGGAGGTCTCTCCCTCCCCCAGAGCCCCTCATGCCACAGTGGTACTCTGTGTCTGGCAGGCTACTCTGCCCACCCCAGCATCAGCACAGCTCTCCTCCTCCATCTCAGACTGTGGAACCAAAGCTGGCCCAGTTGTCCATGACAAAAGAGGCTTTTGGGCCAAAATGTGAGGGTGGTGGGTGGGATGGGCAGGGAAGGAATCCTGGTGGAAGTCTTGGGTGTTAGTGTCAGCCATGGGAAATGAGCCAGCCCAAGGGCATCATCCTCAGCAGCATCGAGGAAGGGCCGAGGAATGTGAAGCCAGATCTCGGGACTCAGATTGGAATGTTACATCTGTCTTTCATCTCCCAGATCCTGGAAACAGCAGTGTATATTTTTGGTGGTGGTGGGTTTGGGGTGGGGAAGGGAAGGGCGGGCAAGGAGTGGGGAGGGAGTCTGGGGTGGGAGGGAGGCATCTGCATGGGTCTTCTTTTACTGGACTGTCTGATCAGGGTGGAGGGAAGGTGAGAGGTTTGCATCCACTTCAGGAGCCCTACTGAAGGGAACAGCCTGAGCCGAACATGTTATTTAACCTGAGTATAGTATTTAACGAAGCCTAGAAGCACGGCTGTGGGTGGTGATTTGGTCAGCATATCTTAGGTATATAATAACTTTGAAGCCATAACTTTTAACTGGAGTGGTTTGATTTCTTTTTTTAATTTTATTGGGAGGGTTTGGATTTTAACTTTTTTTAATGTTGTTAAATATTAAGTTTTTGTAAAAGGAAAACCATCTCTGTGATTACCTCTCAATCTATTTGTTTTTAAAGAAATCCCTAAAAAAAAAAATTATCCAATTGAACGCACATAGCTCAATCACACTGGAAATGTTTGTCCTTGCACCTGAGCCTGTTCCCACTCAGCAGTGAGAGTTCCTCTTTGCCCTGAGGCTCAGTCTCTCTCGTATTTTGTCCCCACCCCCAATTCCTTGAGTGGTTTTTGCTCTAGGGCCCTTTCTTGCACTGTCCAGCTGGTTGTACCCTCTCCAGGCATTTATTCAACAAATGTGGGTGAAGTGCCTGCTGGGTGCCAGGTGCTGGGAATACATCTGTGGACAAGACATGCTTGGGTCCTACTCCTGGAGCACTGTAAAAAGAGCTGATTCAAGTAAGTAGATGCCTGTTTTGAGACCAGAAGGTTTCATAATTGGTTCTACGACCCTTTTGAGCCTAGAATTATTGTTCTTATATAAGATCACTGAAGAAAGAGGAACCCCCACAACCCCCTCCACAAAGAGACCAGGGGCGGGTGATGAGACCTGGGGTTTAGAACCCCAGGTGAGACCTCAAATCACTGCATTCATTCTGAGCCCCCTTCCTGTCCCCAGGGGAGGTGTATTGTGTATGTAGCCTTAGAGCATCTCTGCCTCCAACCCAGCAGTTCTCTGCCAAAGCTTGTGGAGGAGGGAGAGCCCTGTCCCTGCCCTCAGGCTCCCCAGTGCTCCTGGCCCTTCTATTTATTTGACTGATTATTGCTTCTTTCCTTGCATTAAAGGAGATCTTCCCCTAACCTTTGGGCCAATTTACTGGCCACTAATTTCGTTTAAATACCATTGTGTCATTGGGGGGACCGTCTTTACCCCTGCTGACCTCCCACCTATCCGCCCTGCAGCAGAACCTTGGCGGTTTATAGGTAATGATGGAACTTAGACTCCTCTTCCCAGAGTCACAAGTAGCCTCTGGGATCTGCCAACACACGTCCACTCCCAAGCCACTAGCCCACTCCCCAGTTGGCCCTTCTGCCCTTACCCCACACACAGTCCAACTCTTCCACCTCTGGGGAAGATGGAGCAGGTCTTTGGGAAGCTCCCACACCCACCTCTGCCACTCTTAACACTAAGTGAGAGTTGGGGAGAAACTGAAGCCGTGTTTTTGGCCCCCCGAGGCTAACCCTGATCCATAGTGCTACCTGCACCTCTGGATTCTGGATTCACAGACCAAGTCCAAGCCCGTTCTTACGTCGCCATAAAGGCCCCCGAACGGCATTCTCGGTACTTCTGTTTGTTTTTGTACATTTTATTAGAAAGGACTGTAAAATAGCCACTTAGACACTTTACCTCTTCAGTATGCAAATGTAAATAAATTGTAATATAGGAAATCTTTTGTTTTAATATAAGAATGAGCCTGTCCAATTTCTGCTGTACATTATTAAAAGTTTTATTCACAGA
